# A computer vision-based system for recognition and classification of Urdu sign language dataset

**DOI:** 10.7717/peerj-cs.1174

**Published:** 2022-12-14

**Authors:** Hira Zahid, Munaf Rashid, Sidra Abid Syed, Rafi Ullah, Muhammad Asif, Muzammil Khan, Amenah Abdul Mujeeb, Ali Haider Khan

**Affiliations:** 1 Biomedical Engineering Department and Electrical Engineering Department, Ziauddin University, Karachi, Pakistan; 2 Electrical Engineering Department and Software Engineering Department, Ziauddin University, Karachi, Pakistan; 3 Biomedical Engineering Department, Sir Syed University of Engineering and Technology, Karachi, Pakistan; 4 Optimizia, Karachi, Pakistan; 5 Electrical Engineering Department, Ziauddin University, Karachi, Pakistan; 6 Biomedical Engineering Department, Ziauddin University, Karachi, Pakistan

**Keywords:** Urdu sign language, Sign language, Pattern recognition, SVM, KNN, Random Forest, Bag of words

## Abstract

Human beings rely heavily on social communication as one of the major aspects of communication. Language is the most effective means of verbal and nonverbal communication and association. To bridge the communication gap between deaf people communities, and non-deaf people, sign language is widely used. According to the World Federation of the Deaf, there are about 70 million deaf people present around the globe and about 300 sign languages being used. Hence, the structural form of the hand gestures involving visual motions and signs is used as a communication system to help the deaf and speech-impaired community for daily interaction. The aim is to collect a dataset of Urdu sign language (USL) and test it through a machine learning classifier. The overview of the proposed system is divided into four main stages *i.e.*, data collection, data acquisition, training model ad testing model. The USL dataset which is comprised of 1,560 images was created by photographing various hand positions using a camera. This work provides a strategy for automated identification of USL numbers based on a bag-of-words (BoW) paradigm. For classification purposes, support vector machine (SVM), Random Forest, and K-nearest neighbor (K-NN) are used with the BoW histogram bin frequencies as characteristics. The proposed technique outperforms others in number classification, attaining the accuracies of 88%, 90%, and 84% for the random forest, SVM, and K-NN respectively.

## Introduction

Sign language plays a vital role in bridging the gap between deaf people communities, and between deaf people and non-deaf people, sign language is widely used. Sign language involves the usage of different parts of the body, such as fingers, hand, arm, head, body, and facial expression (Cheok, Omar & Jaward, 2019). There are five main parameters in sign language, which are hand-shape, palm orientation, movement, location, and expression/non-manual signals. To have an accurate sign word, all of these five parameters must be performed correctly (Rastgoo, Kiani & Escalera, 2021). Hand gestures in sign language can be either static or dynamic. Alphabets and numbers from 0–9 in USL and ASL are static gestures, whereas words and sentences require the combination of dynamic gestures and facial expressions. Advancements in sign language research and technology have led to applications that can ease daily life interactions for the deaf community. These include translation systems, interpreting services, video remote human interpreting, human–computer interaction (Deng et al., 2017), as well as online hand tracking of human communication in desktop environments (Tagliasacchi et al., 2015). Hand gesture research has led to applications in the gaming world such as (Lee et al., 2021) where gestures were recorded on a leap motion controller to play a whack-a-mole game, and Virtual Reality (VR), and Mixed Reality (MR) technology, as new interactive personal computers. Sign language communication additionally changes in various nations and districts. A large number of serious nations have set up their sign language communications. America, China, and Arabic nations are the most significant nations which have their sign language communications (Agarwal & Thakur, 2013).

Approximately 3.3 million Pakistanis are suffering from any kind of disability of which 0.24 million are impaired hearing which approximates to 7.4% of the overall disabled. A very important point is that 55% of the total disabled lie in the age group from 5–29 years (Habibullah et al., 2021). As discussed by Akram & Bashir (2015), at present, the Directorate General of Special Education in Pakistan runs 56 institutions focused on the education and rehabilitation of children with special needs. The complete dataset for USL alphabets has been compiled and made public access in Imran et al. (2021). Much research is still required to create more efficient SLRS (sign language recognition systems) for USL and methods to implement these models in both educational environments and in daily life. This research aims to create a standardized dataset for USL numeric (0–9) and models a recognition system. However, in Pakistan, this cycle is under scrutiny. In Pakistan, unimpaired people face a lot of problems communicating with deaf people due to the lack of SL understanding, learning resources, and interpreters. In Pakistan, numerous organizations are attempting to grow more standard sign-language communication, yet at the same time, they cannot collect a reviewed model (Alvi et al., 2007). USL is in the development phase. Researchers in the field of sign languageare working to develop a more centralized and standardized SL in Pakistan. USL is not fully developed yet. Different organizations are working on developing USL, but still, according to researchers’ best knowledge, they are unable to compile a standardized version (Abid et al., 2014). Due to the lack of machine learning data in USL, to extend the framework of USL and to facilitate the deaf in communication there is a need for a technique that could translate sign language inreal-time.

The goal of this study is to build an automated method for recognizing USL numbers based on the bag-of-words (BoW) histogram characteristics. Gathering data through USL counting should be the first step. BoW-based histogram bin centers are employed for these vocabulary terms during feature extraction. To create a BoW-based histogram for feature extraction, the image’s pixel intensities are mapped to the closest vocabulary. To get the feature vector, the bin frequencies of the histogram are employed. Classifiers such as kNN, SVM, and random forest are used for classification in the last step of the algorithmic process.

## Related Work

Analysis of feelings in Urdu proceeds at its initial maturity compared with other languages rich in the capital such as English. In comparison, the work was limited such that the amount of surveys and analysis papers already published was explicitly influenced. Anwar, Wang & Wang (2006) submitted a description of the strategies that rely on the creation of Urdu corpus in their analysis of automated Urdu language processing. Different linguistic techniques such as language tagging, parsing, and recognition by named entities were employed. In the early surveys on Urdu language therapy, there was a shortage of the necessary techniques for conducting sentimental research in Urdu, which is discussed in this report. Studies focusing on Urdu SA have been few and far between. This can be put down to the lack of interest from language engineering entities and the shortage of linguistic resources. For the most part, past studies conducted on the Urdu language emphasized the various aspects of language processing (Anwar, Wang & Wang, 2006; Khattak et al., 2021). Much of the audience is not conscious of PSL since the knowledge differences between the two classes are immense. Likewise, the deaf cannot interpret written documents in English and Urdu. The provision of an appropriate translation model will also help a deaf community’s cognitive capacity to comprehend natural language. In order to convert phrases written in English into corresponding PSL phrases, a grammar-dependent converter model is recommended. This is a first attempt to use the main NLP techniques for the conversion of every natural language to PSL. The method suggested includes a systematic procedure for investigating the PSL linguistic framework and formulating the PSL word grammar structure. Thus these rules become context-free grammar, which can then efficiently be used as the parsing module in which target PSL sentences are translated and validated (Khan, Abid & Abid, 2020; Anwar, Wang & Wang, 2006). In addition to that, there are a few commercially accessible Urdu OCR typed text systems but no framework for Urdu handwritten text is accessible. It is important to notice that handwritten text recognition is named ICR in the field of simulation and design recognition whereas printed text identification is recognized as OCR (optical character recognition). The text uses ICR for the identification of manuscript text (Wali & Hussain, 2007). Sign language (SL) is an additional universal language that the deaf population knows and adopts worldwide.

Although SL is a medium for communication, in reality, most people also do not interpret SL correctly, so it is a communication challenge again between an individual who has discomfort with speech and listening problems and regular persons. In this respect, the various study tries to overcome this problem with wearable devices and other analytical paradigms. However, most of them were concentrated on the English or Western languages which gave the citizens of the Indian sub-continent a trivial resolution. In this article, we suggest a way to resolve this problem by utilizing a Kinect Motion Sensor, which can be interpreted and listened to in the natural language by means of hand motions and expressions, for the interpretation of signals (SL). The framework suggested is built on a practical basis, so we saved signs in a dictionary/training package for baseline creation (Sohail et al., 2020). In the study of Urdu handwritten text in the literature survey, it has been observed that many models of machine learning, for example, SVM (support vector machines) (Cortes & Vapnik, 1995), NB (naive Bayes) (Sagheer et al., 2010), and ANN (artificial neural network) (McCulloch & Pitts, 1943). In the study of text-photos, we also demonstrated the competitiveness of these methods. Many scholars in the literature suggest using CNN (LeCun et al., 1989) to retrieve details from the photographs comprising text data. Moreover, impressive work has shown that CNN is one of the most widely utilized DNNs in the production of images during dynamic activities such as matching patterns, object analysis, and so on. Furthermore, without advanced awareness of syntactic (or semantical) language constructs, CNN is equally relevant to the data corpus at a word or character stage. In different data science-related functions, from vision applications to machine applications to speech recognition and others, the CNN paradigm is equally relevant. The explanation for the continuing use of DNN is the right mathematical relationship that the given input and output requirements, whether linear or not, regardless of the underlying design of the model. In addition, the knowledge in DNN traverses the layers that measure each output likelihood. DNN is therefore one of the most effective and consistent solutions to the above-mentioned activities. The capacity to isolate and classify unique characteristics through deep-learning models often leads significantly to incisive and accurate study outcomes. They have also been seen to compete with standard styles. The Urdu handwritten text recognition literature (Ahmad et al., 2017) also proposed profound network models to produce maximum performance within a limited span of time.

A procedure that transforms a collection of 32 binary number 25 variations that represent five finger positions in UP and DOWN numbers to decimal numbers. Palm removal, practical point removal process, training step and test phase are used for the conversion of binary numbers. These techniques may be used to transform the static and dynamic pictures. Then, using the binary decimal algorithm, the binary numbers shall be transformed into decimal numbers. Then, 12 vowels, 18 consonants, and one Aayutha Ezhuthu are transformed into Tamil numbers. If checked through a static picture recognition method, 96.87 percent of the pictures out of 320 have been correct. When the real-time (five times) of 32 images is concerned, we get 160 images in the dynamic recognition process. All the photos show the right result except two signals, which is 98.75% of the exact symbol identity (Rajam & Balakrishnan, 2012). A leap motion interface may be used to build an advanced two-way contact mechanism for all auditory impediments. Disabled individuals will disappear with their world. Management has an integrated absorption stage to control the LMC monitoring by analyzing finger activity in real-time. For identification of hand motion, they use Secret Markov Model. The definition applauds a modern form of communicating by integrating the identification of hand motion with speech transition. They developed an android app for speech/text conversion with the aid of the Google API and have many required functionality such as emergency calls and position monitoring for treatment purposes (Gul et al., 2020). Nasir et al. (2014) propose a voice interpreter artificial sign language that starts by recording the 3D video stream by Kinect, and then focuses on the joints of concern in the human skeleton. In communicating the message in Pakistani sign language, the suggested framework deals with the issues facing mute citizens. The 3D trajectory algorithm is used to process the normalized results. This analysis. The acts conducted are listed with the comprehensive ensemble learning technologies. The movements are transformed into expression until understood. This device was checked on many PSL signs showing how realistic it is to use ASLSI in real-time. First tests have been carried out with ASL since support was readily accessible, but we switched to PSL soon. Few important variations between ASL and PSL were found that were resolved at this point and modules modified accordingly. The standard deviation was 8.89, the average accuracy is 86%. Furthermore, Kanwal et al. (2014) contributes the first ever attempt in terms of fabrication of Pakistani Sign Language translating glove which is portable as well as cost-effective. As the sensor values from the glove vary from person to person, this system was made to use pattern recognition approach. In order to accomplish the task, Principal Component Analysis (PCA) was employed for feature extraction and Euclidean distance as classification technique. At this time, the system has ten static gestures in its library and it perfectly judges nine gestures out of targeted ten gestures which are commonly used in Urdu sign language. Furthermore, a summarized literature review has also been mentioned in the Table 1.

**Table 1:** A mini literature review of the dataset that are based on Urdu language mini literature review.

**Author/Year**	**Dataset name**	**Dataset type**	**Dataset demographics**	**Publically available or not?**	**Overall accuracy**
Chandio et al. (2020)	NA	Text images from environment	NR	Yes	95%; 78.13%
Imran et al. (2021)	NA	Signs of 37 characters of Urdu language	NA	Yes	90%
Sagheer et al. (2009)	CENPARMI	Images of s isolated digits, numeral strings with/without decimal points, five special symbols, 44 isolated characters, 57 Urdu words (mostly financial related)	NR	No	97%
Ahmad et al. (2017)	UPTI	Images of sentences	NR	Yes	96%; 95%
Khan et al. (2020)	NA	26 alphabets in USL using sEMG	NA	No	81%; 63%
Arafat & Iqbal (2020)	CLE	Synthetic image with ligature	NR	Yes	94%
Ahmed et al. (2019)	UNHD	Sentences of Urdu language	500 writers	Yes	92%
Proposed dataset	NA	Sign images of 0-9 Urdu counting	104 male and female participants	No	88%; 90%; 84%

**Notes.**

Abbreviations NA Not available CENPRMI Center for Pattern Recognition and Machine Intelligence UPTI Urdu Printed Text Image CLE Center for Language Engineering UNHD Urdu Nastaleeq Handwritten Dataset

In Table 1, a mini literature review of the datasets has been conducted and the table indicates the only one dataset by Imran et al. (2021) is based on images of USL and that too only contain alphabets not counting. The proposed dataset contains sign images of 0–9 Urdu counting.

## Dataset

### Collection methods

We placed a 6-inch tripod stand on a table and fixed a Samsung Galaxy J4 cellphone into it. A white paper was taped to the wall opposite the table, which acted as our screen and background for this dataset. The cellphone’s camera was magnified to 3.4 mm, so it could zoom into the screen. This allowed focus only on the screen and no other distractions were present. We showed sample images of the USL dataset to 104 volunteers. The volunteers were asked to replicate the signs, onto the white screen. We corrected their hand posture and adjusted their hand onto the centerline of the screen. Volunteers were asked to use only their right hands for this dataset. All signs in USL are right-handed. The images were clicked by one of the authors while another author directed the sequence of the signs formed by the volunteers. Numbers 0, 1, 2, 3, 4, and 5 were captured using both dorsal and palmar sides of the right palm. Numbers 6, 7, 8, and 9 were captured in only one direction. 15 images were taken from each volunteer. After capturing a set of images from a volunteer, the images were viewed by both authors to find any inconveniences. These included blurry images, incorrect hand posture, or realignment of hands with the center of the screen. Such images were deleted and captured again with the same volunteer. This ensured a fair and reasonable dataset with set parameters. A detailed specifications of the proposed dataset are presented in Table 2 and come images from the dataset are presented in Table 3.

**Table 2:** Specification of the proposed dataset specs.

**Parameters**	**Proposed dataset**
Aspect ratio	Square
Type of data	Image file
How data were collected	Numerous orientations and forms photographs (hand configuration) are taken using a mobile camera.
Data format	Raw
Image size	Variable
Image format	RGB
Hands used for signs	1 hand used (right side only)
Static signs	Palms images
Lighting conditions	Uniform lighting
Background conditions	Uniform background
Total number of signs	10
Number of images per participant	15
Number of images from palmar side	5
Number of images from dorsal side	10
Number of participants	104
Total no. of images	1,560
Choice of participants	Students, Teachers/Instructors
Focal length	4mm
Brand of camera	Samsung
Camera model	SM-J400F
Flash mode	No flash
Max aperture	1.85
Data source location	Karachi, Sindh, Pakistan

**Table 3:**
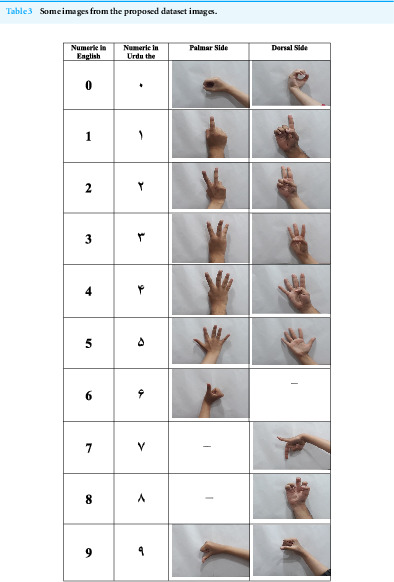
Some images from the proposed dataset images.

## Methodology

Any image processing method begins with pre-processing and segmentation. Instead of processing the whole picture, only a portion of it is processed. The complete image processing system would impose an undue strain on the image processing system for image recognition. We have a wide range of segmentation methods, and several of the popular segmentation methods are discussed here. Picture segmentation may be local segmentation, which is primarily linked to the identification of a single region of the image, or global segmentation, where the focus is on the whole image, consisting of a vast number of pixels. Images meant for USL identification may suffer from low feature quality if there include too many noisy background pixels. As a result, a preprocessing step is required to minimize noise and remove background noise. To decrease noise, an additional picture is recorded in the background and removed from the original. The resultant image is then filtered using a median filter. To make the feature extraction process as straightforward as possible, the filtered picture is transformed to grayscale. USL number recognition is projected to increase as a result of preprocessing, which improves hand motion photos and minimizes background noise.

### Feature extraction

When dealing with a huge dataset, extraction becomes a difficult process for ASL number recognition because of the reliance on the extracted feature’s quality for an effective solution. This study, on the other hand, provides a BoW-based feature extraction approach that uses pixel intensities to build histograms for several pattern recognition issues (Wang et al., 2013). To begin, ten percent of the pictures in the BoW vocabulary training collection are randomly selected for each class. All the BoW vocabulary for each class is gathered together into a single dictionary;

(1) \begin{eqnarray*}D=\bigcup _{i=1}^{N}Vi.\end{eqnarray*}

With N being the total number of classes examined in Eq. (1). When creating a BoW-based histogram from an image, the vocabulary in D is used as the histogram-bin centers in both the training and testing stages (Bhuiyan et al., 2021). As a result, a histogram that accounts for every pixel in each sign picture may be generated. USL number recognition is based on the frequency of BoW histogram bins in this article. The pixel intensities of each grayscale picture are utilized to extract the bin centers or to generate histograms in this suggested BoW-based feature extraction method. The final histogram-based feature extraction might employ pixel intensity triple as feature points if the supplied sign pictures are in color.

### Classifiers

#### K-nearest neighbor (kNN)

The kNN is a widely used, basic, non-parametric supervised classifier (Zhang et al., 2019; Gou et al., 2019). It forecasts the category of a test feature using a proximity search to determine the K closest training neighbors. The ‘Cityblock’, ‘Cosine’, ‘Correlation’, and ‘Euclidean’ distance functions are often used to calculate the distances between the new test feature and all the training data in the feature set. The test sample is labeled with the label that contains the large percentage of the K nearest training samples. To determine an appropriate value for K, values in the range of 1 till 4 were tried and however, the optimal results with *k* = 1. In the proposed methodology, we have used the Euclidean distance formula (Eq. (2)) to classify the images with the value of *k* = 1.

(2) \begin{eqnarray*}d \left( p,q \right) =\sqrt{\sum _{i-1}^{n}({q}_{i}-{p}_{i})^{2}}\end{eqnarray*}

where *q* and *p* are two points, *q*_*i*_ and *p*_*i*_are vectors and *n* is the space.

#### Support vector machine (SVM)

The support vector machine (SVM) is an old classification technique that has grabbed scientists’ attention, notably in the disciplines of machine classification, regression, and learning. SVMs are related to well-defined classes. The term “feature extraction” or “filtering” refers to this process. Even when no prediction of unknown samples is required, function selection and SVM classification have been utilized in conjunction. They may be used to indicate the primary sets involved in class differentiation. By mapping the entry space, the SVM covers a broad region. The SVM may determine the border of regions belonging to both classes by computing an ideal hyperplane separation. The hyperplane is constructed in such a way that the distance between the nearest workout samples is maximized. By applying multiple kernel functions, the SVM method may produce a variety of learning machines. SVMs are rapidly gaining popularity as a machine learning method for classification, regression, and identification of new objects. They perform well on a wide variety of real-world issues, and the method is logically motivated (Rani & Holi, 2014). The variables of the hyperplane approach are a quadratic optimization problem (Shmilovici, 2009). To label a dataset, do the following:

(3) \begin{eqnarray*} \left( \mathbi{x}1,\mathbi{y}1 \right) \ldots \ldots \left( \mathbi{xn},\mathbi{yn} \right) ,\mathbi{xi}\in \mathbi{Rd}\end{eqnarray*}

where *xi* is a vector representation of a characteristic and y_i_ is a class mark (negative or positive) of a practice formula *i*. The optimal hyperplane is then described as follows

(4) \begin{eqnarray*}\mathbi{wxT}+\mathbi{b}=0\end{eqnarray*}

where *w* denotes the weight matrix, *x* denotes the input vector, and *b* denotes the bias *W* and *b* must satisfy the following in equalities for all components of the training collection.

(5) \begin{eqnarray*}\mathbi{wxT}+\mathbi{b}\geq +1\mathbi{ifyi}=+1\end{eqnarray*}

(6) \begin{eqnarray*}\mathbi{wxT}+\mathbi{b}\leq -1\mathbi{ifyi}=-1.\end{eqnarray*}

The primary goal is to find *w* and *b* in order to form a hyperplane and increase the margin. Support-vectors are vectors *xi* for which —*yi*— (*wxiT* + *b*) = 1.

#### Random forest

Regression and classification issues may be solved using a random forest, a machine learning approach. Ensemble learning, a method that combines several classifiers to solve complicated problems, is used. Many decision trees are used in a random forest method. Random forests are trained *via* bagging or bootstrap aggregation, which creates a ‘forest’. Machine learning algorithms may be improved by using a meta-algorithm called bagging. Based on the predictions of the decision trees, the random forest algorithm determines the result. The no. of estimators/trees used in the random forest is 500. The Gini impurity function has been employed while training the data for the algorithm (Khan et al., 2021). Mean or average output from different trees is used to make predictions. Increased accuracy is achieved by using more trees. A random forest method eliminates the drawbacks of a decision tree model. It improves precision while decreasing dataset overfitting. Predictions are generated without the need for a large number of parameters in packages (ScienceDirect, 2022).

### Experimental setup

**Figure 1:**
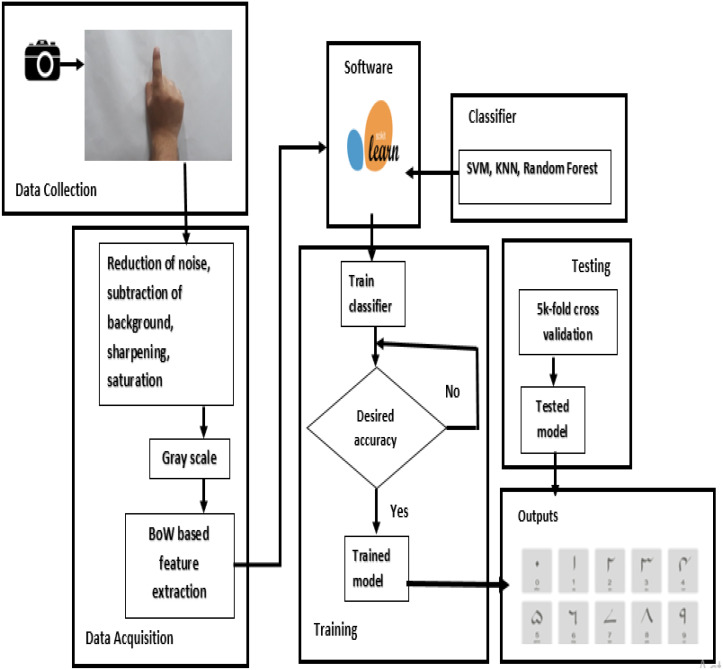
Block diagram representation of the proposed methodology.

The images are reduced reasonably and are converted to grayscale so that overall data is reduced to a reasonable size. Images were placed in folders. Folder name has been used to label the images. The input images have been reduced to different sizes by using the program developed in Python. The parameters on which results have been taken are the number of images in the dataset, the number of the epoch, the size of the images (which ultimately affects the input layer), and the size of the filters used. The input is a raw image *i.e.*, pixels, the pixels are converted to edges, edges into corners and contours, then into object parts and ultimately classified into the output category. Figure 1 shows the detailed methodology of the proposed approach in the current study.

## Results

**Figure 2:**
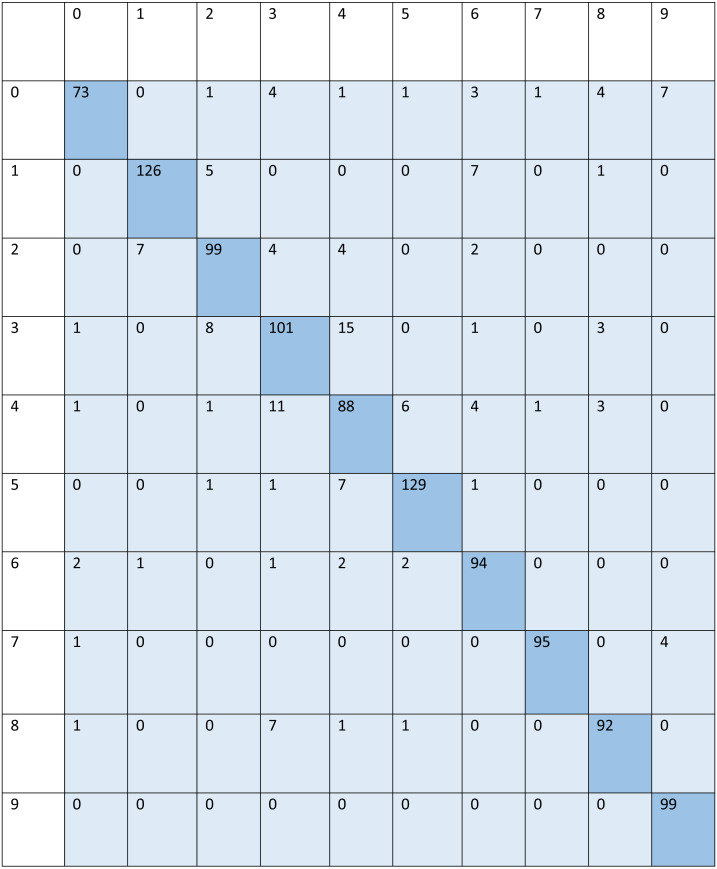
Confusion matrix of random forest.

**Figure 3:**
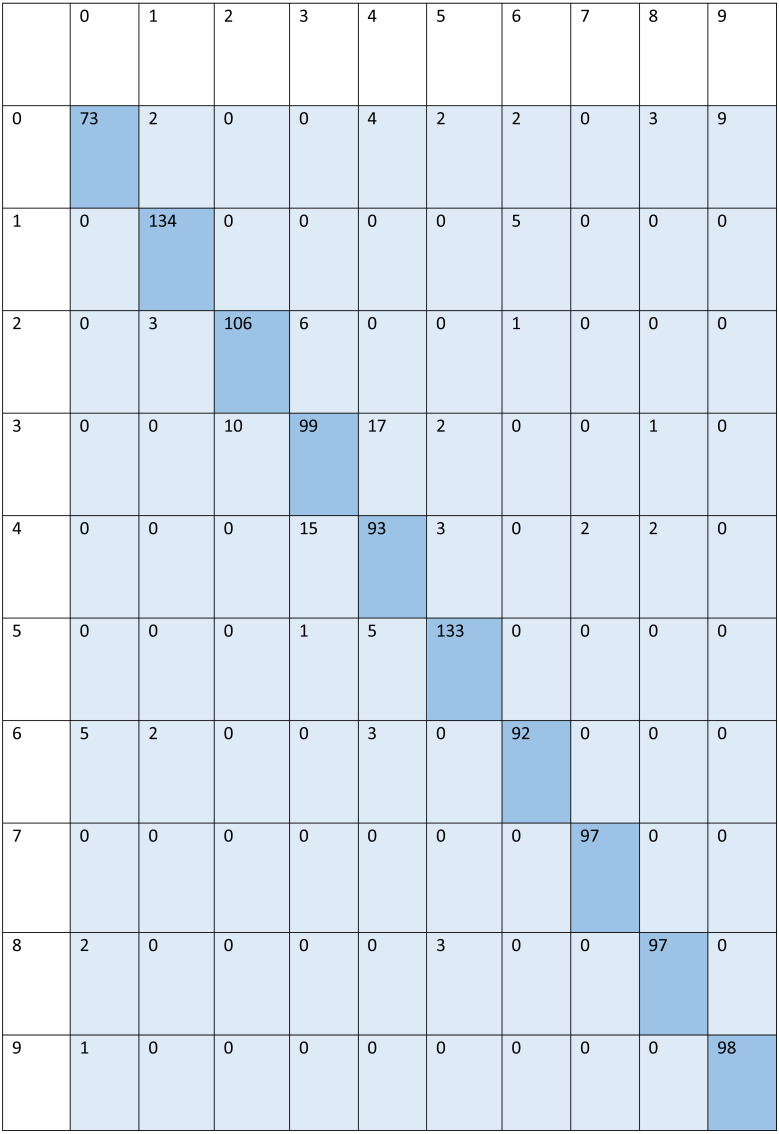
Confusion matrix of SVM.

**Figure 4:**
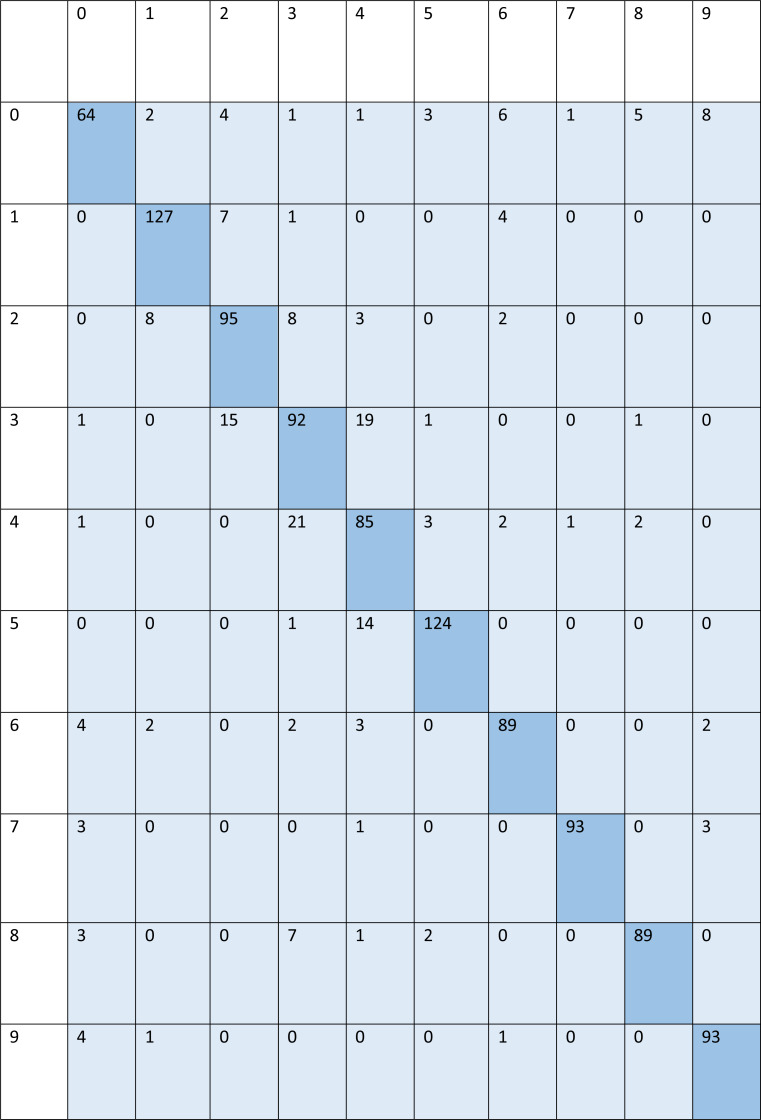
Confusion matrix of kNN.

For signed pictures, classification yields four distinct forms of recognition. It is possible for an image pertaining to one class to be incorrectly classified as belonging to another class, which results in a false positive (Fp) or false negative (Fn) identification of that class, respectively. Recognition is characterized as either a true positive (Tp) or a true negative (Tn) depending on how well an image’s class can be anticipated. Accordingly, the suggested algorithm’s performance is evaluated using the following standard metrics: accuracy, precision, recall, and F1 score. After training and testing data of 1,560 images, the achieved average accuracy of 88% for the random forest, 90% for SVM, and 84% for kNN respectively. Figures 2–4 shows the confusion matrix graphs of the random forest, SVM, and kNN respectively. Table 4 shows the weighted accuracy, precision, and F1-score of SVM, kNN, and random forest. Tables 5–7 shows precision, recall, and F1-score values of the random forest, SVM, and kNN calculated through Eqs. (8)–(10) respectively.

(7) \begin{eqnarray*}Accuracy& = \frac{{T}_{P}+{T}_{n}}{{T}_{P}+{T}_{n}+{F}_{p}+{F}_{n}} \end{eqnarray*}

(8) \begin{eqnarray*}Precision& = \frac{{T}_{p}}{{T}_{P}+{F}_{P}} \end{eqnarray*}

(9) \begin{eqnarray*}Recall& = \frac{{T}_{P}}{{T}_{P}+{F}_{n}} \end{eqnarray*}

(10) \begin{eqnarray*}F1~score& = \frac{2\times (\Pr\nolimits \times \mathrm{Re})}{PR+Re} \end{eqnarray*}

where *PR* stands for precision and Re stands for Recall in Eq. (10).

## Discussion

**Figure 5:**
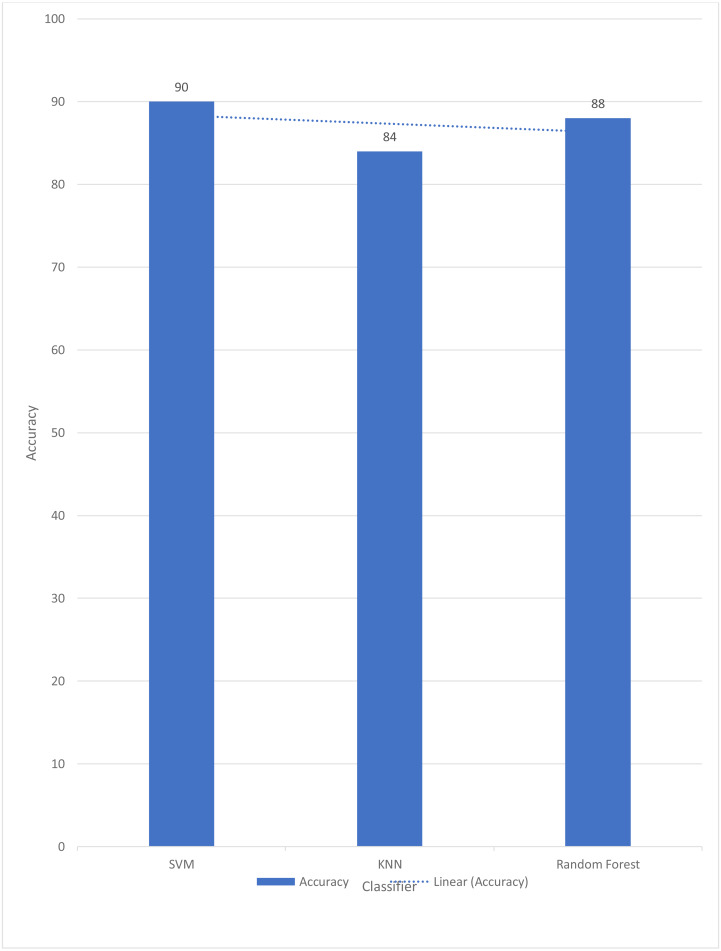
Graph of accuracies with trend achieved by the proposed methodology.

In Table 4, the weighted accuracies, precision, and F1 score are reported of all three classes. And it is clearly indicated that SVM reports the highest accuracy. Several literatures reports the use of linear SVM reports the highest accuracies in sign language (Jain et al., 2021; Ekbote & Joshi, 2017; Mali, Limkar & Mali, 2019; Katoch, Singh & Tiwary, 2022). SVM maps data to a high-dimensional feature space so data points may be classified even when not linearly separable. The data are converted so the category separator may be represented as a hyper plane. SVM classifiers are accurate when given a clear separation margin and high-dimensional space. Figure 5 shows the comparison graph of all three classifiers.

**Table 4:** Accuracy of SVM, kNN, random forest.

**Classifier**	**Accuracy**	**Weighted precision**	**Weighted recall**	**Weighted F1-score**
Random forest	0.88	0.88	0.88	0.88
SVM	0.90	0.90	0.90	0.90
K-NN	0.84	0.84	0.84	0.84

**Table 5:** Precision, Recall and F1-score values of random forest.

	**Precision**	**Recall**	**F1-score**
0	0.92	0.77	0.84
1	0.94	0.91	0.92
2	0.86	0.85	0.86
3	0.78	0.78	0.78
4	0.75	0.77	0.76
5	0.93	0.93	0.93
6	0.84	0.92	0.88
7	0.98	0.95	0.96
8	0.89	0.90	0.90
9	0.90	1.00	0.95
Macro average	0.88	0.88	0.88
Weighted average	0.88	0.88	0.88

**Table 6:** Precision, Recall and F1-score values of SVM.

	**Precision**	**Recall**	**F1-score**
0	0.90	0.77	0,83
1	0.95	0.96	0.96
2	0.91	0.91	0.91
3	0.82	0.77	0.79
4	0.76	0.81	0.78
5	0.93	0.96	0.94
6	0.92	0.90	0.91
7	0.98	0.97	0.97
8	0.94	0.95	0.95
9	0.89	0.99	0.94
Macro average	0.90	0.90	0.90
Weighted average	0.90	0.90	0.90

**Table 7:** Precision, Recall, and F1-score values of kNN.

	**Precision**	**Recall**	**F1-score**
0	0.80	0.67	0.73
1	0.91	0.91	0.91
2	0.79	0.82	0.80
3	0.69	0.71	0.70
4	0.67	0.74	0.70
5	0.93	0.89	0.91
6	0.86	0.87	0.86
7	0.98	0.93	0.95
8	0.92	0.87	0.89
9	0.88	0.94	0.91
Macro average	0.84	0.84	0.84
Weighted average	0.84	0.84	0.84

A similar approach was opted by Bhuiyan et al. (2021). It is proposed in this study that the automated identification of American Sign Language (ASL) numbers be accomplished using a model based on a bag-of-words (BoW). The BoW-based characteristics have been demonstrated to be particularly useful for categorizing USL numbers in experiments. K-NN, SVM, and random forest are applied for classification. In order to classify ASL numbers, the suggested histograms are proven to have distinguishing properties. BoW histogram bin frequencies are used as characteristics for the K-nearest neighbors (K-NN) classifier, which classifies based on those frequencies. Big-scale tests on two large ASL number-recognition datasets are used to validate the proposed technique, which achieves an F1 score of 99.92% on the Kaggle ASL digits dataset when categorizing the digits. But in the current study, authors opted for SVM because SVM works well when there is a clear line between different classes. SVM is better at working in places with a lot of dimensions. SVM is good when the number of features is bigger than the number of samples, so it can be used. There are many ways to classify things in machine learning, but SVM is better than most of them because it has better results. The space of the boundary that separates the two classes. That it can also work in n-Dimensional space. Whereas in graph 1, it can also be seen that random forests also perform better comparatively than K-NN because the random forest can also handle large datasets with tens of thousands of variables. When a class is less common than some other classes in the data, it may instantly equalize the data sets.

## Conclusion

In this research, an effective USL number identification technique was constructed by extracting characteristics from histograms of bag-of-words (BoW). This dataset was used to test the proposed scheme’s accuracy, precision, recall, and F1 score. The BoW-based histogram approach beat both traditional and BoW-based histogram techniques on all performance indicators, as shown by these data. For the random forest, the accuracy was 88%; for the SVM, it was 90%; for kNN, it was 84%. Deaf and mute persons will be able to interact with others through intelligent gadgets utilizing hand gestures, and the gaming and robotics sectors will benefit as a result. The authors want to use semi-supervised learning approaches to expand the suggested BoW-based model for ASL number identification to a bigger dataset where categorizing all pictures is difficult. In the future, the author plans to add more images to the dataset and make it more diversified by adding USL alphabets.

##  Supplemental Information

10.7717/peerj-cs.1174/supp-1 Supplemental Information 1 Code

## List of Abbreviations

USL: Urdu Sign Language
SLRS: Sign Language Recognition Systems
SVM: Support Vector Machine
KNN: K-Nearest Neighbor
CENPARMI: Center for Pattern Recognition and Machine Intelligence
UPTI: Urdu Printed Text Image
CLE: Center for Language Engineering
UNHD: Urdu Nastaleeq Handwritten Dataset

## Additional Information and Declarations

### Data Availability

The following information was supplied regarding data availability: The data is available at Zenodo: Hira Zahid, Munaf Rashid, Sidra Abid Syed. (2022). A Computer Vision-based System for Recognition and Classification of Urdu Sign Language Dataset. https://doi.org/10.5281/zenodo.7326179.
